# Intelligent flexibility in an aging society: lessons from Japan's home-visit nursing system

**DOI:** 10.3389/fpubh.2026.1755718

**Published:** 2026-02-24

**Authors:** Takemasa Ishikawa, Haruka Imura, Yoshiyuki Takashima

**Affiliations:** 1 Nana-r Home-visit Nursing Development Center, Tekix Corporation, Toyonaka, Japan; 2 Graduate School of Biomedical and Health Science, Hiroshima University, Hiroshima, Japan; 3 Faculty of Nursing, School of Medicine, Nara Medical University, Kashihara, Japan

**Keywords:** community care, health system governance, home-visit nursing, Japan, long-term care

## 1 Introduction

The home-visit nursing system constitutes the cornerstone of Japan's policy response to the rapid population aging and declining fertility rates. Introduced under medical insurance in 1992 for older adults and expanded to all age groups in 1994, the system was further extended by launching the Long-Term Care Insurance (LTCI) scheme in 2000 (1, 2). It was designed to support home-based living, contract hospital capacity, and reduce family caregiving. Subsequent reforms extended coverage to people with disabilities, mental illnesses, and rare diseases, creating one of the most inclusive community nursing frameworks in high-income countries (3). While many high-income nations provide home healthcare, Japan's model is distinct in its integration of medical, long-term care, and welfare services within interconnected insurance schemes (2, 4). This is a unique example of a comprehensive insurance-based community nursing governance.

Policy instruments, such as reimbursement revisions, special home-visiting nursing instructions, and inter-scheme coordination, have been gradually layered to respond to new social and clinical demands (3). These mechanisms have enabled the flexible delivery of home-visit nursing across the medical, long-term care, and welfare sectors, integrating diverse functions from acute symptom management to end-of-life care.

However, this adaptability increases system complexity. Multiple overlapping schemes have blurred administrative responsibilities and created variations in service access (5). Understanding how Japan's home-visit nursing system functions, how it supports an aging society, and where its institutional boundaries fall is critical for future health system design. This Opinion aims to clarify how Japan's multi-layered home-visit nursing system balances flexibility and governance, and to discuss future directions for designing responsive and sustainable community-care frameworks. This perspective examines the strengths and challenges of a system built to create a balance between flexibility, inclusiveness, and sustainability. This article introduces the concept of intelligent flexibility to examine how Japan's home-visit nursing system has adapted to demographic aging under institutional complexity. The following sections describe the institutional development of Japan's home-visit nursing system, examine governance challenges arising from its flexibility, and discuss the implications of intelligent flexibility for future community care.

## 2 Institutional design and evolution

Within Japan's healthcare system, home-visit nursing operates under two main insurance frameworks: medical insurance and LTCI. Medical insurance covers patients requiring ongoing medical management, typically limited to three visits per week, whereas LTCI provides nursing within a point-based budget determined by certified care need levels (3). The LTCI provides care within a fixed benefit limit based on assessed care-need levels, supporting budgetary stability and interdisciplinary planning. However, when patients have high medical needs but relatively low care-need certification levels, or when multiple long-term care services are used concurrently, this structure can constrain the frequency and duration of nursing visits (6). The coexistence of these two frameworks ensures adaptability but creates administrative complexity in determining which scheme applies to each case. In many Western countries, home healthcare operates within a single framework, such as the NHS in the United Kingdom and Medicare in the United States (4). These systems offer administrative simplicity but limited adaptability across domains.

In contrast, Japan's multi-layered framework has evolved through gradual policy adaptations that allowed nurses to work across medical and long-term care boundaries while accepting administrative complexity as a trade-off for flexibility. Each institutional mechanism was created to address specific service gaps, thus expanding the scope of nursing beyond conventional medical and long-term care boundaries. Together, these mechanisms enable the provision of nursing care across acute, chronic, and end-of-life stages (3).

At the acute end of the continuum, the Special Home-Visiting Nursing Instruction (Tokubetsu Homon Kango Shijisho) allows physicians to authorize intensive nursing visits for patients whose conditions temporarily require heightened medical management or closer clinical monitoring, such as during an acute exacerbation or when intensive wound care for deep pressure ulcers is needed. This arrangement demonstrates the system's regulated form of flexibility, balancing clinical responsiveness with administrative control (3). Beyond acute care, additional institutional layers were introduced to support long-term and highly dependent populations. Appendix 7 specifies disease categories, such as amyotrophic lateral sclerosis, progressive muscular dystrophy, and Parkinson's disease-related disorders. This arrangement allows for more frequent and longer nursing visits, sometimes exceeding 4 days per week or involving multiple visits per day (3). Although LTCI generally takes precedence for users certified for long-term care, Appendix 7 allows medical insurance to be applied even to LTCI-eligible individuals, and Appendix 8 permits extended or frequent visits when high medical dependency warrants it (3). Psychiatric home-visit nursing also forms part of this multi-layered structure and currently accounts for approximately one-third of all nurse-provided medical insurance home-visit nursing days (7).

Welfare-based subsidy programs, such as the Self-support Medical Care and the Medical Care Subsidy for Specified Intractable Diseases, reduce or cap the out-of-pocket costs for eligible patients. Table 1 summarizes these frameworks and illustrates how Japan's flexibility has evolved through gradual and problem-oriented policy adjustments. Together, these mechanisms form an adaptive, though increasingly complex, structure that enables nurses to deliver care across the medical, long-term, and welfare sectors.

**Table 1:** Institutional mechanisms supporting the flexibility and inclusiveness of Japan's home-visit nursing system.

**Institutional mechanism**	**Legal/administrative basis**	**Primary target population**	**Main function**	**Key limitation**
Special home-visiting nursing instruction (Tokubetsu Homon Kango Shijisho)	Medical insurance; physician-issued instruction valid up to 14 days.	Patients experiencing a temporary increase in medical needs or unstable clinical conditions.	Allows physicians to authorize time-limited intensive nursing visits when heightened medical management or closer clinical monitoring is required.	Valid for up to 14 consecutive days Generally issued once per month. May be issued twice per month for patients with tracheostomy or pressure ulcers requiring intensive wound care.
Appendix 7 (Beppyo 7)	Medical insurance.	Patients with specific designated diseases (e.g., ALS, muscular dystrophy, Parkinson's disease, spinal cord injury).	Allows long-term, continuous home-visit nursing under medical insurance regardless of long-term care certification.	Exempt from weekly visit-frequency limits Subject to general medical-insurance rules and payer-specific administrative review
Appendix 8 (Beppyo 8)	Medical and LTCI.	Patients requiring continuous medical management, such as those receiving home oxygen therapy, home hemodialysis, total parenteral nutrition, pressure-ulcer care, home pain management, or treatment for pulmonary hypertension.	Supports ongoing home nursing for medically dependent patients requiring continuous management. No limit on reimbursable visits.	LTCI prioritized if certified Fragmented state-based eligibility.
Self-support medical care program (Jiritsu Shien Iryo)	Welfare-based public subsidy under the Services and Supports for Persons with Disabilities Act; the psychiatric outpatient component is administered under the Act on Mental Health and Welfare for Persons with Mental Disorders.	Patients certified under the program categories (rehabilitative medical care, medical care for children, or psychiatric outpatient care) who require specified medical treatments or assistive devices. Home-visit nursing may be covered when applicable.	Reduces copayments through public assistance (income-based caps), thereby maintaining continuity of necessary medical services, including home-visit nursing when eligible.	Means-tested with municipal application Periodic renewal Coverage and copayment limits vary by income category and certification type.
Medical care subsidy program for specified intractable diseases	Public medical expense subsidy under the Act on Medical Care for Patients with Intractable/Rare Diseases (Specified Intractable Diseases Act); administered by MHLW.	Patients certified with designated intractable diseases or childhood chronic specified diseases eligible for public subsidy.	Reduces copayments for approved medical services, including home-visit nursing when specified in the treatment plan, thereby improving the affordability of continuous care for patients with rare and intractable diseases.	Eligibility and copayment caps vary by disease category and income level Administrative certification required.
Psychiatric home-visiting nursing	Introduced in 2010 under medical insurance; expanded in 2013. Requires a Psychiatric Home-Visiting Nursing Instruction issued exclusively by a psychiatrist.	Patients with mental illness, developmental disorders, or other psychiatric conditions requiring community-based care.	Provides home-based psychiatric nursing care under a psychiatrist's instruction, integrating mental health support into the home-care framework.	Instruction can be issued only by psychiatrists Nurses require psychiatric nursing experience or complete designated training. Workforce remains limited, with regional disparities in service availability.

ALS, amyotrophic lateral sclerosis; MHLW, Ministry of Health, Labor and Welfare; LTCI, long-term care insurance.

This table summarizes key institutional mechanisms that respond to diverse medical and social needs through layered insurance arrangements in Japan. “Appendix 7” and “Appendix 8” refer to official schedule categories defined in Japan's medical and long-term care insurance regulations, rather than supplementary appendices to this manuscript.

## 3 Challenges of flexibility and governance

Among high-income countries, Japan's model is unusually inclusive; however, its inclusiveness has created inconsistencies and areas of under-coverage. Because eligibility and reimbursement differ between the medical and LTCI systems, access to services may depend as much on institutional classification as on clinical needs. Patients with chronic conditions such as heart failure or chronic kidney disease may have substantial medical requirements but limited access to home-visit nursing if their functional impairment is mild; they are classified primarily under the LTCI system, which restricts the number and duration of visits within the LTCI care-need budget (3, 8). Conversely, patients with medical insurance may receive more frequent or longer visits, resulting in differences in care intensity, even among patients with comparable needs (2, 9).

The coexistence of multiple schemes renders it difficult for the system to navigate. Families are required to navigate complex eligibility rules, documentation requirements, and boundaries between medical and social care. For many patients, this complexity delays care initiation and causes premature discontinuation (10).

A similar challenge is observed among providers, who must interpret overlapping administrative guidance and negotiate with different insurers. These procedural demands consume staff time and contribute to regional variations in how services are organized and reimbursed (5, 11). Flexibility within the system sometimes encourages agencies to pragmatically interpret rules to ensure patient access. However, this can blur accountability and introduce variations in billing and service patterns. The inclusiveness that strengthens adaptability may also produce subtle disparities, where patients' access to appropriate home nursing depends partly on providers' coordination capacity and the administrative interpretation of eligibility, rather than on need alone. Evidence indicates that home-visit nursing may also mitigate caregiver burden and psychological distress among family members, suggesting broader social inclusiveness (12).

The institutional flexibility that characterizes Japan's home-visit nursing system has supported responsiveness to diverse needs but has also introduced governance challenges. The coexistence of multiple reimbursement routes and exceptions has blurred the boundaries between clinical appropriateness and resource allocation. Under the medical insurance scheme, frequent or extended visits can be reimbursed at higher rates than those provided under the LTCI. Some agencies may prioritize medical insurance billing when it is clinically permissible, as the reimbursement levels are higher than those under LTCI. This potential incentive arising from differences in reimbursement rates between the two schemes has been acknowledged in government discussions on appropriate service allocation (13). This structural incentive may lead to overlapping expenditures and inefficient public fund allocations (14). In countries with centralized governance structures, such as the United Kingdom's Care Quality Commission or the Centers for Medicare and Medicaid Services in the United States, auditing and oversight are more standardized (4). However, in Japan, the governance of home-visit nursing is largely decentralized, leaving boundary management and compliance to individual providers.

This challenge is not limited to the financial aspects. In a context where home-visiting nurses operate autonomously across settings, differences in clinical judgment or documentation standards may lead to variations in care intensity and frequency (15). Agencies with stronger administrative capacities secure stable revenues, whereas smaller rural providers struggle to meet documentation and audit requirements (10). This imbalance creates an uneven field of operation, which can reinforce geographic disparities in access.

Policymakers have periodically attempted to restore consistency through fee revisions and auditing systems. However, these adjustments often add to procedural complexity. The Japanese experience exemplifies a broader dilemma regarding adaptive healthcare systems. Flexibility improves inclusiveness and timeliness, but weakens regulatory coherence. Balancing professional discretion with standardized accountability remains a central issue in sustainable home-visit nursing. Taken together, these findings describe how institutional flexibility has evolved in Japan's home-visit nursing system, while also revealing its governance limitations.

## 4 Discussion: intelligent flexibility in home-visit nursing

For decades, the system has relied on the discretion of nurses and managers to navigate the complex eligibility and reimbursement criteria. Such human-mediated flexibility allows tailored care, but makes system performance dependent on each agency's administrative and interpretive capacity. As demographic pressures intensify and the nursing workforce ages, this dependence becomes unsustainable (16). In this context, intelligent flexibility refers to a mediating system capacity that links individual-level needs with service provision through data-informed mechanisms.

Future governance will require what may be described as intelligent flexibility, a structure that maintains responsiveness while ensuring transparency and consistency through digital, data-driven mechanisms. The growing use of standardized electronic documentation and eligibility verification tools can help minimize the variations in administrative interpretations among insurers and providers (17). Data linkages between the medical and long-term care sectors would also enable policymakers to monitor gaps in service coverage, identify duplications of visits, and forecast regional workforce needs with greater accuracy (1). However, as Japan's medical and long-term care services operate under separate insurance schemes with distinct payment systems and data repositories, integrating data across these two domains has been difficult. Consequently, integrated research linking the two sectors has progressed slowly despite their interdependence in practice (18). Current evidence on the effectiveness of Japan's home-visit nursing system remains largely observational, with a limited number of randomized trials. Embedding standardized outcome measures and data linkages can strengthen causal inferences and support evidence-based governance (19).

Operationally, artificial intelligence is increasingly used as a tool to support providers' and policymakers' decision-making and coordination in home and community care. Recent studies have illustrated such applications. Soltani et al. (20) used a long short-term memory model to forecast palliative home hospitalization demand, while Huang et al. (21) combined Andersen's behavioral model with machine learning to identify older adults likely to require medical services. These approaches could help allocate home-visit nursing resources more efficiently by identifying patients who may need earlier or more frequent visits. The goal is not to eliminate discretion, but to make it visible, auditable, and collectively learnable across agencies. Intelligent flexibility represents the shift from a system that relies on experience and negotiation to one that learns from data. Embedding such learning mechanisms within Japan's complex framework could not only reduce inefficiencies but also help identify individuals who might otherwise be overlooked by the current system.

Emerging evidence suggests that Japan's home-visit nursing is associated with fewer hospital admissions and more home deaths, although findings on quality of life and caregiver burden remain mixed (19, 22). Japan's home-visit nursing system offers an instructive example for other aging societies seeking to balance inclusiveness, flexibility, and accountability in community-based care. Japan's incremental layering shows that large-scale adaptation can occur without major reforms through gradual problem-oriented adjustments. This incremental approach has enabled Japan to sustain service continuity across diverse populations, ranging from children with disabilities to frail older adults. This example should not be interpreted as a transferable institutional model. Japan's multi-layered insurance arrangements reflect country-specific contexts. The transferable lesson lies in the governance logic: using data-informed mechanisms to mediate between individual needs and system-level resource allocation.

However, Japan's experience also shows the limitations of flexibility: without coherence, incremental measures can fragment rather than integrate care. For other countries adopting multischeme models, coherence, data transparency, and equity are essential.

More broadly, Japan's experience reframes how healthcare systems can adapt to super-aging societies. The goal is not simply to expand coverage but to enable systems to learn, identify unmet needs, optimize resources, and refine governance through feedback loops. A well-calibrated, data-informed framework can maintain a people-centered orientation of care while rendering flexibility measurable and accountable, thus providing a potential model of sustainable community care for the twenty-first century. Japan's approach suggests that flexibility must be supported by coherent governance and transparent data systems in order to remain sustainable as societies age.
